# Norwegian version of the Edinburgh cognitive and behavioural ALS screen: Construct validity, internal consistency, inter-rater, and test-retest reliability

**DOI:** 10.1371/journal.pone.0285307

**Published:** 2023-05-04

**Authors:** Tina Taule, Irmelin Smith Eide, Line Fjær, Mari-Anne Myrberget, Marit Sofie Oseland, Marit Arnevik Renså, Tone Revheim, Ole-Bjørn Tysnes, Jörg Aßmus, Tiina Rekand

**Affiliations:** 1 Department of Occupational Therapy, Orthopedic Clinic, Haukeland University Hospital, Bergen, Norway; 2 Department of Rehabilitation Services, Haraldsplass Deaconess Hospital, Bergen, Norway; 3 Department of Physio and Occupational Therapy, Namsos hospital, Nord-Trøndelag Hospital Trust, Namsos, Norway; 4 Department of Clinical Services, St. Olav’s University Hospital, Trondheim, Norway; 5 Department of Social Work, Occupational Therapy and Physiotherapy, Hospital of Southern Norway, Kristiansand, Norway; 6 Department of Neurology, Neurologic Clinic, Haukeland University Hospital, Bergen, Norway; 7 Centre for Clinical Research, Haukeland University Hospital, Bergen, Norway; 8 Institute of Neuroscience and Physiology, Sahlgrenska Academy, University of Gothenburg, Gothenburg, Sweden; Houston Methodist Academic Institute, UNITED STATES

## Abstract

**Background:**

Research collaboration highlight a need for validated tests in other languages than English. Translation and culture adjustments may threaten essential features of the original instrument.

**Objective:**

To assess the internal consistency, inter-rater and test-retest reliability, and construct validity of the Norwegian version of the Edinburgh Cognitive and Behavioural Amyotrophic Lateral Sclerosis (ALS) Screen (ECAS-N).

**Methods:**

Performance of 71 subjects with ALS, 85 healthy controls (HC) and 6 controls with Alzheimer’s disease (AD) were assessed with the ECAS-N. Test-retest interval was four months. Internal consistency was evaluated using Cronbach’s alpha; reliability was assessed using intraclass correlation coefficient (ICC), Cohen’s kappa, and Bland Altman plot. Five hypothesis, including the Montreal Cognitive Assessment (MoCA) screen, was evaluated for construct validity.

**Results:**

ECAS-N total score produced a Cronbach’s alpha of 0.65, had excellent inter-rater reliability (ICC = 0.99) and acceptable test-retest reliability (ICC = 0.73). Construct validity analysis suggested valid use of the ECAS-N to distinguish people with ALS-specific cognitive impairment from HC (p = 0.001) and those with AD (p = 0.002). The MoCA and ECAS-N were moderately correlated (r = 0.53).

**Conclusion:**

The ECAS-N has potential to be used by different testers in clinical practice and research to screen patients with ALS who speak Norwegian and for documenting cognitive impairment over time.

## 1. Introduction

Amyotrophic lateral sclerosis (ALS) is a neurodegenerative multisystem disease that may entail cognitive and/or behavioral changes in addition to deleterious changes in motor function [1, 2]. The presence of cognitive and behavioral symptoms in ALS can precede motor symptoms [3] and may increase caregiver burden [4]; shorten lifespan [5, 6]; and lower adherence to treatment recommendations [7]. ALS is the third most common neurodegenerative disease, an adult onset disorder, and is characterized by rapid progression [8].

As a first step to detect declines and manage ALS challenges, early and repeated screening of possible cognitive and behavioral changes is recommended for ALS-specific healthcare [9]. During the decade, the Edinburgh Cognitive and Behavioural ALS Screen (ECAS) has proven to be an appropriate and reliable test, as it can measure the heterogeneity of cognitive and behavioral changes in ALS, and in addition takes into account substantial motor difficulties that are common in ALS [9–12]. The ECAS has been translated and adapted for clinical and research use in Asia [13, 14]; Europe [15–23]; including Norway [24].

As with all translated assessment instruments, translation of the ECAS depends on accounting for language and culture peculiarities of the target language. Failure to recognize this important factor may threaten the equivalence between the original and translated version [25]. Confidently evaluating patient results also depends on a valid translation [26]. What specific parameters are important? A cultural understanding of hypothetical concepts may differ among different demographic groups, and the meaning attached to each translated item may diverge across test subjects or administrators of different groups. In addition, test conditions and population characteristics may vary among countries. For each translated scale, trustworthiness must be established that the individual items of the translated test perform in the same way as the original test (internal consistency). In a validated test, one must also establish that test scores are not affected by who administers the test (inter-rater reliability). Additionally, for patients in a stable state, their scores must also be stable over time (test-retest reliability). Knowledge about how a test performs in a variety of situations (construct validity) will help expand the possibilities of test score interpretations [26].

As measurement properties can vary and are not a fixed feature, our study aimed to assess the reliability and construct validity of the version of the ECAS translated into Norwegian (ECAS-N). The study addressed the following research questions in patients with ALS: (a) What is the internal consistency of items in the ECAS-N? (b) What is the inter-rater reliability of the ECAS-N when administered by different testers? (c) What is the test-retest reliability of the ECAS-N for a test-retest interval of 4 months? (d) What is the construct validity of the ECAS-N when compared against the Norwegian version of the Montreal Cognitive Assessment (MoCA), and the ECAS-N results of healthy controls (HCs) and persons with Alzheimer’s disease (AD)? The ECAS-N and the MoCA are both tests of cognition, assessing similar, but not the same cognitive domains [27, 28]. While the ECAS is specifically designed for people with ALS, the MoCA is a generic test of cognition [27, 28], meaning the two tests are expected to have different sensitivity to ALS-specific cognitive problems.

For construct validity, we hypothesized that: (H1) ECAS-N and the MoCA scores for persons with ALS would be moderately positively correlated; (H2) ECAS-N scores of subjects with ALS and HCs would show a strong negative association; (H3) ALS-specific scores for subjects with ALS would be higher compared to subjects with AD; and (H4) ALS non-specific scores for subjects with AD would be higher compared to subjects with ALS; (H5) ECAS-N scores of subjects with ALS and subjects with AD would be moderately positively associated.

## 2. Material and methods

To ensure transparent reporting, we report our studies according to the Strengthening the Reporting of Observational Studies in Epidemiology (STROBE) [29].

### 2.1. Design

A validation design [30] was used to determine selected psychometric properties of the ECAS-N. This design comprise specific scientific steps to estimate the sample size, distinct statistical analyses to assess the degree to which the scores of the ECAS-N are free from measurement error (reliability) and assesses the degree to which the ECAS-N truly measure the psychological construct it purports to measure (validity) [30].

### 2.2. Protocol and registration

Before any study procedures were carried out with subjects, the protocol was published [31], and the study was registered at ClinicalTrials.gov (registration number NCT03579017; available at https://clinicaltrials.gov/ct2/show/NCT03579017).

### 2.3. Participants

Inclusion criteria for ALS subjects, HCs and controls with AD are listed in Table 1.

**Table 1:** Inclusion and exclusion criteria for subjects with ALS and control subjects.

Criteria		Subjects with ALS	Healthy controls	Controls with AD
Inclusion characteristics				
	Native Norwegian speaker	X	X	X
	Between 35–85 years old		X	X
	MMSE-NR3 score, ≥20–25			X
	Clock drawing test score, ≤3			X
Exclusion characteristics				
	Great difficulties in writing or reading	X	X	X
	Comorbidity specific to decline in cognitive function	X	X	X

Abbreviation: AD, Alzheimer’s disease; ALS, amyotrophic lateral sclerosis, MMSE-NR3, Mini Mental Status Examination-Norwegian Revised version number 3.

#### 2.3.1. Persons with ALS

Patients with ALS were eligible to participate if they were followed up at an ALS unit at Haukeland University Hospital (HUH), St.Olavs Hospital, Hospital of Southern Norway or Namsos Hospital within four months after being diagnosed with ALS. A member of the ALS-specific healthcare team recruited study participants between May 2017 and June 2021, and in conjunction with patients’ first visit to the hospital. Each ALS participant also chose one carer to assist them in the study.

#### 2.3.2. Control subjects

Testers recruited healthy volunteers from various places of employment and social organizations. Persons with AD were recruited from Haraldsplass Deaconess Hospital, a hospital with a research group focusing on AD and other types of dementia. AD patients were diagnosed according to standard guidelines used in Europe [32]. To ensure that all participants themselves were able to make an informed decision about study participation, we included only patients with mild-moderate AD. All volunteers who fulfilled the inclusion criteria (see Table 1) and agreed to participate were enrolled. We aimed to closely match the control subjects’ age and sex to those in the ALS group. Control subjects were between the ages of 35 and 85 years. We strived to have approximately equal distribution of the number of subjects for each group below and above 60 years old. Slightly more male control subjects were selected to match the sex distribution of the ALS group. The numbers of subjects for each group with lower or higher levels of education were also approximately equal.

### 2.4. Data collection

We used a custom-designed questionnaire to collect subjects’ age, gender and level of education. Patients were assessed with the ECAS-N using country-specific cutoff scores [24], and the Norwegian version of MoCA (version 7.1; available at www.mocatest.org).

#### 2.4.1. The ECAS

The ECAS is a brief, multi-domain screening test designed to detect and document ALS patients’ cognitive impairment (ECAS-cognitive screen) and behavioral changes (ECAS-behavioral screen) [27]. The cognitive screen comprises an ALS-specific subscore (verbal fluency, language and executive functions including social cognition); an ALS-nonspecific subscore (memory and visuospatial functions); and a total score. The total ECAS-cognitive screen score ranges from 0–136, the ALS-specific subscore from 0–100, and the ALS-nonspecific subscore from 0–36. For all cognitive scales lower scores indicate greater deficits. The ECAS-behavioral screen is a carer interview designed to assess and document patient performance changes in five domains of behavior and three domains of psychosis. Behavioral scores range from 0–10, and scores for psychosis changes range from 0–3. For behavioural and psychosis scale, higher scores indicate greater deficits. Systematic reviews have shown that the ECAS is an effective, valid and reliable screening test of cognitive changes in patients with ALS [11, 33].

ECAS performance can be assessed either by obtaining spoken responses or written responses from the subjects in order to accommodate physical disabilities that often accompany ALS [27]. The ECAS was translated into Norwegian language and culture by use of the scientific procedure described by Sousa et al. [34].

#### 2.4.2. The MoCA

The MoCA is a brief screening tool used to assess impairment in frontal lobe function and mild cognitive impairment. It assesses performance in the following domains: visuospatial/executive, naming, memory, attention, language, abstraction, delayed recall, and orientation [28]. The MoCA has been validated in patients with ALS [35]. The maximum possible score is 30, with scores below 26 indicative of cognitive impairment. Performance of subjects with 12 year of education or less is compensated by adding 1 point to the subject’s total MoCA score [28].

#### 2.4.3. Testing procedures

Assessment were conducted between May 2017 and October 2021. For patients with ALS and their carers, baseline performance on the ECAS-N and MoCA tests was obtained within four months of ALS diagnosis, and the retests were done within eight months after being diagnosed. The time interval between baseline and retest administration was considered long enough to minimize recall bias but short enough to reduce the possibility of study withdrawal due to symptom worsening. Previous research also provided evidence that the constructs measured by the tests are stable over our study period [36, 37].

The ECAS-N cognitive screen was administered either in the subjects’ homes, in an outpatient clinic, or during hospitalization. The ECAS-N behavioral screen was administered either through a face-to-face interview or by asking the questions over the phone. Decisions about the testing modality depended on established routines for each hospital, the wish of the test subjects, and recommendations given by local authorities regarding the Covid-19 pandemic. Eight individual testers, all of them specially trained for administering the ECAS-N, administered and scored the tests. All participants were given the same instructions, following ECAS guidelines [38]. Baseline tests and retest were conducted by the same tester.

Procedures for assessing inter-rater reliability on the ECAS-N involved two testers independently scoring the participants at the same time. Due to the extra cost and effort of this reliability assessment, only subjects from one hospital contributed data to it. Four of the testers were involved in these scorings.

### 2.5. Sample size estimation

For evaluation of the measurement properties relevant in our study, Terwee et al. recommend a minimum of 50 participants [39].

### 2.6. Statistical analyses

Data were analysed using SPSS version 26.0 [IBM 40] and Matlab version 9.0 [41]. Descriptive statistics were used to summarize group demographic variables and selected baseline characteristics for subjects with ALS and control subjects.

#### 2.6.1. Internal consistency

We used the Cronbach’s alpha statistic to estimate the internal consistency among the subscales and the internal consistency for the total scale of the ECAS-N. A Cronbach’s alpha coefficient of 0.70 is a commonly considered to be good internal consistency [39].

#### 2.6.2. Inter-rater reliability and agreement

For continuous variables, inter-rater reliability was assessed with the intraclass correlation coefficient (ICC), which reflects both the degree of correlation and agreement between measures [42]. The ICCs and their 95% confident intervals (CIs) were estimated by using a two-way random-effects analysis of variance model with the interaction term for assessing the absolute-agreement between single raters. We used this two-way random-effects model, since the ECAS is designed for routine clinical use by any therapist trained as a selected rater in this reliability assessment [43]. Single scores reflect that a single rater usually completed the ECAS assessment and it was chosen to avoid calculating a misleading high ICC. As the patients’ cognitive status was to be judged according to ECAS-N cutoff scores, absolute agreement was needed.

There is no consensus on how to interpret the magnitude of the ICC. We considered ICC values equal to 0.70 or better to be acceptable [39]. Additionally, we used the 95% CI of the estimated ICC as the basis for ICC interpretation [39, 43]. For dichotomous variables of the ECAS-N (cognitive screen), we used Cohen’s kappa statistic [44] to determine if there was agreement between the two testers regarding judgement of patients as being cognitive impaired or not. Kappa values greater than 0.60 was considered to be acceptable [25].

Inter-rater agreement between two independent testers for total ECAS-N, ALS-specific, and ALS-nonspecific scores was estimated with the Bland Altman method [45, 46]. This analysis is a straightforward way to evaluate if scores derivate from each other–in our case, two independent testers’ scoring of participants–and whether there are systematic differences. Bland Altman procedure present a plot in which systematic errors can easily be seen [45, 46]. For each participant, we calculated the absolute difference between the ECAS-N scores (separately for total, ALS-specific, and ALS-nonspecific scores) obtained by tester one and tester two (y-axis), and plotted this difference against the mean difference of the two scores (x-axis). The upper and lower limits of agreement were calculated using the mean and the SD of the differences between scores of tester one and tester two. For normally distributed data, the scatter should be evenly dispersed along the x-axis, and the mean difference should be close to zero [45, 46]. With this method, 95% of the data points should be within ±2SD of the mean difference [47]. Pre-established acceptable limits of agreement were 7 for total score, 5 for ALS-specific score, and 2 for ALS-nonspecific score.

#### 2.6.3. Test-retest reliability and agreement

For continuous variables, also test-retest reliability was evaluated by calculating the ICC [42]. A two-way mixed-effects analysis of variance model with the interaction for assessing the absolute agreement between single scores is often taken to be the most appropriate for test-retest studies [43, 48]. By using a two-way model we acknowledge that the sequence of testing time was an important factor and that the two assessment times were not interchangeable. Using a mixed-effects model, we considered a fixed effect of time, as it was pre-specified and identical across all study subjects. As for inter-rater reliability (see 2.6.2), single scores was used because the ECAS-N is usually administered by one rater. We decided to use absolute agreement, because we assumed that the construct of interest (cognitive function) was stable for the subjects across the two assessment times.

Again we used the 95% CI of the estimated ICC as the basis for ICC interpretation; ICC values greater than 0.70 were deemed acceptable [39]. For dichotomous variables in the ECAS-N, we used Cohens’s kappa to determine if there were meaningful differences in agreement between baseline and follow-up judgements of patients being cognitive impaired or not. Kappa values greater than 0.60 were considered to be acceptable [25].

Test-retest agreement was further assessed by the Bland-Altman method [45, 46]. For each participant, we calculated the absolute differences between the ECAS-N scores (separately for total, ALS-specific, and ALS non-specific scores) obtained at baseline and retest (y-axis), and plotted this difference against the mean difference of the two scores (x-axis). Limits of agreement were calculated using the mean and the standard deviation (SD) of the differences between baseline and retest scores. As for the inter-rater agreement assessment (see 2.6.2), we used the same pre-established acceptable limits of agreement.

#### 2.6.4. Construct validity

To assess construct validity of the ECAS-N, we used subjects’ baseline ECAS-N scores and their MoCA scores. Five *a priori* hypotheses (H1-H5) were evaluated. Pearson product-moment correlation coefficient were computed to evaluate hypothesis 1. A t-test for independent groups and chi-square test for independence was used to evaluate hypothesis 2–5. P-values less than 0.05 were considered significant.

### 2.7. Ethical considerations

This study was evaluated by the Regional Ethical Research Committee (reference number 2015/1221/REK vest) and approved by the Data Privacy Unit at HUH, Bergen, Norway (reference numbers 2016/3166). All the participants and their carers signed a written informed consent form in compliance with the revised Declaration of Helsinki of 1987 [49].

## 3. Results

**Fig 1:**
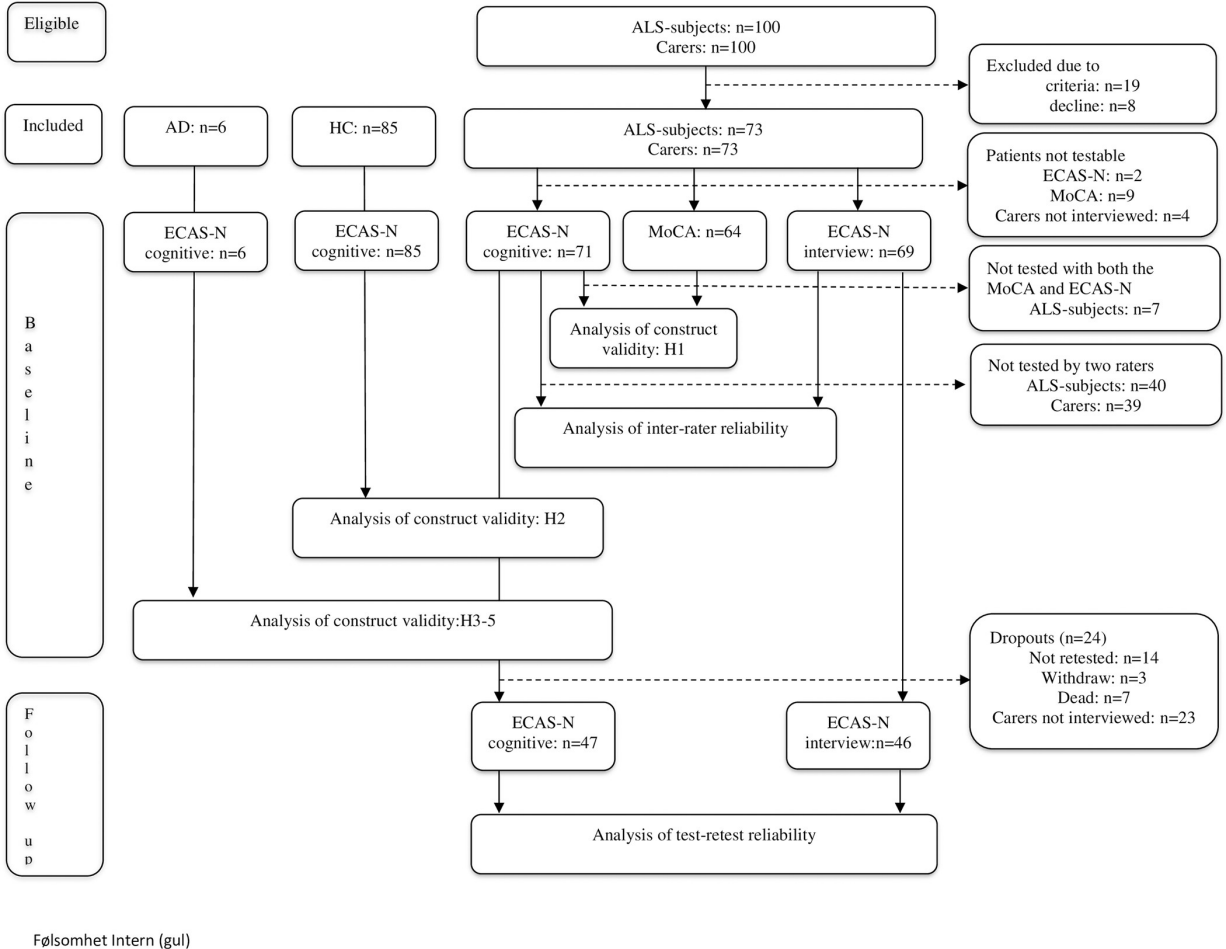
Flow diagram showing selection of study subjects, inclusion/exclusion criteria, test instruments used, and timing of test administration. Abbreviations: AD, Alzheimer’s disease; ALS, amyotrophic lateral sclerosis; ECAS-N, Edinburgh Cognitive and Behavioural ALS screen-translated Norwegian version; H1, hypothesis 1; H2, hypothesis 2; H3, hypothesis 3; H4, hypothesis 4; H5, hypothesis 5; HC, healthy control subjects; MoCA, Montreal Cognitive Assessment.

A flow diagram showing participant selection, inclusion/exclusion criteria, and testing procedure summaries are presented in Fig 1.

### 3.1. Subject demographics

Characteristics of 85 HCs, 6 controls with AD, and subjects with ALS are presented in Table 2.

**Table 2:** Characteristics at baseline for subjects with ALS and control subjects^a^.

Variables		Subjects with ALS				Healthy controls	Controls with AD
		Internal consistency, construct validity (H2-5)	Construct validity (H1)	Test-retest reliability	Inter-rater reliability	Internal consistency, construct validity (H2)	Construct validity (H3-5)
		n = 71	n = 64	n = 47	n = 31	n = 85	n = 6
Gender, female, n (%)		24 (34%)	20 (31%)	10 (21%)	11 (36%)	41 (48%)	3 (50%)
Age, n (%)							
	< 60 y	19 (27%)	18 (28%)	15 (32%)	6 (19%)	42 (49%)	0
Educational level							
	High to elementary school	43 (61%)	38 (59%)	25 (53%)	15 (48%)	42 (49%)	4 (67%)
Dominant hand, n (%)							
	right	61 (86%)	56 (88%)	42 (89%)	27 (87%)	77 (91%)	6 (100%)
	left	9 (13%)	7 (11%)	4 (9%)	3 (10%)	5 (6%)	0
	ambidextrous	1 (1%)	1 (2%)	2 (2%)	1 (3%)	3 (4%)	0
ECAS-N-cognitive score, mean (SD)							
	total	100 (14)	102 (14)	102 (13)	101 (13)	113 (10)	65 (8)
	ALS-specific	74 (12)	75 (11)	76 (11)	74 (11)	82 (9)	51 (8)
	ALS-nonspecific	26 (5)	27 (5)	26 (5)	27 (4)	30 (3)	14 (2)
ALS-specific cutoff score ≤65, n (%)		17 (24%)	14 (22%)	8 (17%)	6 (19%)	4 (5%)	6 (100%)
ALS-nonspecific cutoff ≤24, n (%)		22 (31%)	18 (28%)	14 (30%)	10 (32%)	5 (6%)	6 (100%)
ECAS-total cutoff ≤92, n (%)		23 (32%)	18 (28%)	12 (26%)	11 (36%)	4 (5%)	6 (100%)
ECAS-N-behavioral score, median (min, max)		0 (0, 8)^1^	0 (0, 8)^3^	0 (0,8)^3^	1 (0, 8)^3^		
ECAS-N psychosis score, median (min, max)		0 (0, 2)^1^	0 (0, 2)^3^	0 (0,2)^3^	0 (0, 2)^3^		
MoCA-score, mean (SD)		23 (3)^2^	23 (3)	24 (2)^3^	23 (3)^4^		
MoCA cutoff <26, n (%)		55 (78%)^2^	55 (86%)	41 (87%)^3^	23 (74%)^4^		

^a^ Baseline test is test assessment 1; not all subjects contributed data to each type of assessment, thus explaining the different sample sizes for assessment of internal consistency, construct validity, test-retest reliability, and inter-rater reliability. Abbreviations: AD, Alzheimer’s disease; ALS, amyotrophic lateral sclerosis; ECAS-N, Edinburgh Cognitive and Behavioural ALS screen-translated Norwegian version; H1, hypothesis 1; H2, Hypothesis 2; H3, Hypothesis 3; H4, Hypothesis 4; H5, Hypothesis 5; MoCA, Montreal Cognitive Assessment; SD, standard deviation

^1^missing, 2

^2^missing, 7

^3^missing, 1

^4^missing, 5

Of a total sample of 71 subjects with ALS, 31showed evidence of behavioral changes as rated by their carer in at least one of the five behavioral domains. Of these, 21 subjects met the Strong criteria for ALS with behavioral impairment (ALSbi) [9], and for 19 of these, the impairment was based solely on identified apathy. Eight subjects who met the criteria of ALSbi also scored below the cutoffs of the ECAS-N total score. Five of these scored below the cutoffs of the ALS-specific score. Five ALS subjects exhibited psychotic symptoms, as reported by their carers. Two of the patients may have met the diagnosis of amyotrophic lateral sclerosis-frontotemporal dementia (ALS-FTD), with the presence of behavioral and cognitive impairment together with psychotic symptoms [9].

### 3.2. Reliability

Table 3 shows results for the reliability assessments, specifically internal consistency, inter-rater reliability, and test-retest reliability for the ECAS-N total score and subscale scores for subjects with ALS.

**Table 3:** Internal consistency, inter-rater, and test-retest reliability of the ECAS-N in subjects with ALS.

		Internal consistency		Inter-rater reliability			Test-retest reliability		
ECAS-N (number of items)		n	Cronbach’s alpha	n	ICC^3^ Cohen’s kappa	(95% CI)	N	ICC^4^ Cohen’s kappa	(95% CI)
Cognitive screen									
	Total score (15)	71	0.65	31	0.99	(0.98, 1.00)	47	0.73	(0.56, 0.84)
	ALS-specific score (9)	71	0.62	31	0.99	(0.98, 1.00)	47	0.72	(0.54, 0.83)
	ALS-nonspecific score (6)	71	0.33	31	0.97	(0.94, 0.99)	47	0.61	(0.39, 0.76)
Behavioral screen (10)		69	0.72^1^	30	0.89	(0.79, 0.95)	46	0.71	(0.54, 0.83)
Psychosis screen (3)		69	0.50^2^	30	1.00	(1.00, 1.00)	46	0.17	(-0.10, 0.44)
Cognitive cutoff									
	Total (2)			31	0.85	(0.65, 1.05)	47	0.33	(0.02, 0.64)
	ALS-specific (3)			31	1.00	(1.00, 1.00)	47	0.55	(0.24, 0.86)
	ALS-nonspecific (2)			31	0.92	(0.78, 1.06)	47	0.36	(0.07, 0.65)

Abbreviations: ALS, amyotrophic lateral sclerosis; CI, confidence interval; ECAS-N, Edinburgh Cognitive and Behavioural ALS screen-translated Norwegian version; ICC, Intraclass correlation coefficient

^1^Item E-10 of the ECAS Behavior screen was excluded from the analysis, because the variance was zero

^2^Item 1 of the ECAS-Psychosis screen was excluded from the analysis because the variance was zero

^3^Two-way random-effect analysis of variance model with interaction term for the absolute agreement between single raters

^4^Two-way mixed-effect analysis of variance model with interaction term for the absolute agreement between single measurement.

**Fig 2:**
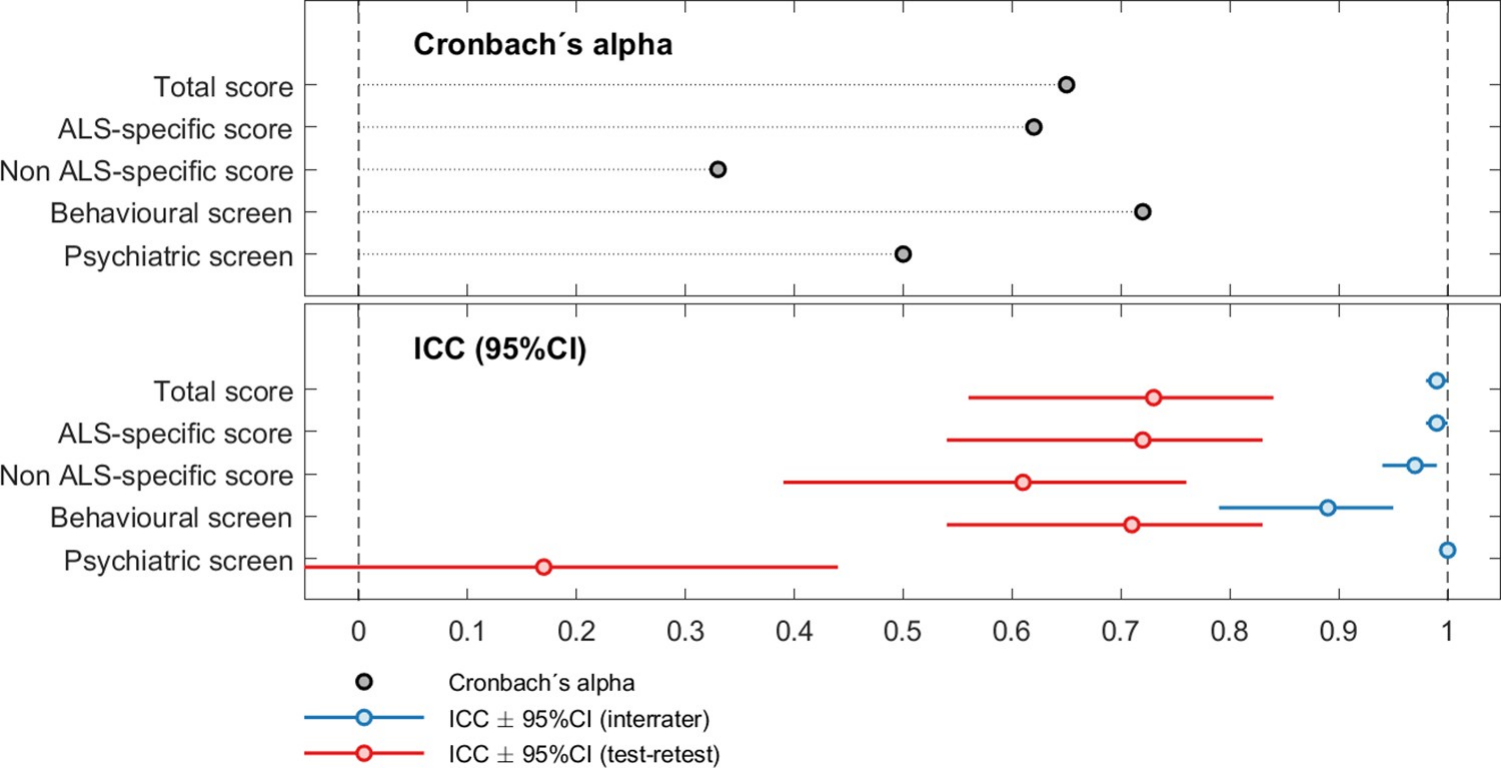
Descriptive visualization of internal consistency (Cronbach’s alpha), inter-rater reliability (Intraclass correlation coefficient, ICC), and test-retest reliability (ICC) of the ECAS-N total score, ALS-specific score, ALS-nonspecific score, behavioural score and pscychiatric screen in subjects with ALS.

A descriptive visualization of reliability of the ECAS-N is provided in Fig 2.

Additional information about reliability and agreement for each specific cognitive domain of the ECAS-N is in the Supplemental files (insert link to S1 Fig and S1 Table here).

### 3.3. Internal consistency

For internal consistency of the ECAS-N using Cronbach’s alpha coefficient (α), we found acceptable values for the ECAS-N behavioral score (α = 0.72). Internal consistency Cronbach alpha values across the other subscales of ECAS-N ranged from α = 0.33 to α = 0.65.

### 3.4. Inter-rater and test-retest reliability

Continuous variable ICCs for interrater reliability ranged from 0.89 to 1.00. Kappa coefficients for categorical variables were above 0.85 for all cutoff scores. Analysis of test-retest reliability using ICCs for continuous variables showed values greater than 0.7 for the ECAS-N total score, ALS-specific score and behavioral score. The ICC was 0.61 for ALS- nonspecific scores. The Kappa coefficient for categorical variables ranged from 0.17 to 0.55.

### 3.5. Inter-rater and test-retest agreement (Bland-Altman)

**Fig 3:**
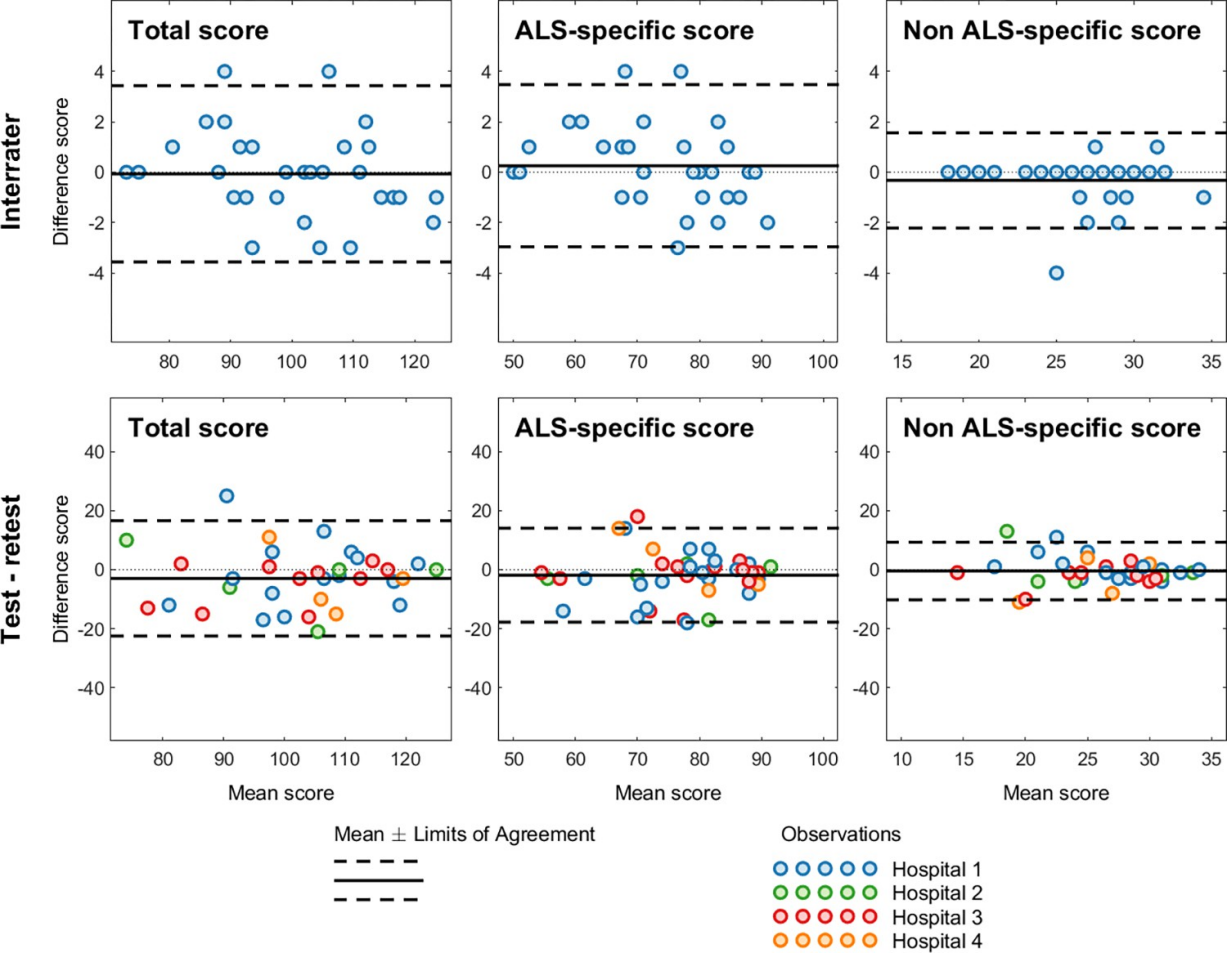
Bland-Altman results for inter-rater agreement of the ECAS-N (top row), and Bland-Altman plot for ECAS-N scores at test time 1 and time 2 (bottom row). The middle solid line represents the mean difference between ECAS-N scores at time 1 and time 2. The lower and upper dashed lines represents the upper and lower 95% confidence limits, respectively.

The Bland-Altman results for inter-rater and test-retest agreement are presented in Fig 3.

The mean difference in ECAS-N total scores between rater 1 and rater 2 was -0.07 (95% CI: -0.71, 0.58); for the ALS-specific score, 0.26 (95% CI: -0.33, 0.85); and for the ALS-nonspecific score, -0.32 (95% CI: -0.67, 0.02). These mean differences were not significantly different from zero for the ECAS-N total score (p = 0.84, SD = 1.75); ALS-specific score (p = 0.38, SD = 1.61); and ALS-nonspecific score (p = 0.07, SD = 0.95). These outcomes indicate that, on average, the ratings of rater 1 and rater 2 were statistically indistinguishable on the ECAS-N. The 95% CIs for agreement were within the predefined acceptable limits (Fig 3). A visual inspection showed that the between rater differences plotted against the mean differences of the inter-rater values was close to zero, and few scores were beyond the upper or lower limits of agreement.

The mean difference between baseline and retest for the ECAS-N total score was

-2.24 (95% CI: -4.70, 0.21); for ALS specific score, it was -1.83 (95% CI: -3.80, 0.13); and for ALS-nonspecific score, it was -0.41 (95% CI: -1.42, 0.60). These mean differences were not significantly different from zero for the total score (p = 0.73, SD = 9.99); ALS-specific score (p = 0.07, SD = 8.00); or the ALS-nonspecific score (p = 0.42, SD = 4.11). This outcome indicates that, on average, the ECAS-N produced the same scores at baseline and retest. For all scores, the 95% agreement interval was beyond the acceptable limits defined *a priori (*Fig 3). Visual inspection indicated that the bias was not severe, because the test-retest differences plotted against the mean differences of the test-retest values was close to zero, and few scores were beyond the upper or lower limits of agreement.

### 3.6. Construct validity (hypothesis testing for H1-H5)

Of the 73 included patients, 2 were unable to complete the ECAS-N and 9 could not complete the MoCA.

In subjects with ALS, we found a moderate positive correlation (r = 0.53) between ECAS-N and MoCA scores, supporting H1. In the testing of H2, we found that ECAS-N total scores, ALS-specific score, ALS-nonspecific score and cutoff scores in subjects with ALS were significantly worse than HCs (see Table 4), supporting H2.

**Table 4:** Comparison of ECAS-N scores in subjects with ALS with HC and those controls with AD.

Variables		Subjects with ALS	Healthy controls	Group difference	Controls with AD	Group difference
		n = 71	n = 85	p-value	n = 6	p-value
ECAS-N-cognitive score, mean (95% CI)						
	Total	100 (97, 103)	113 (111, 115)	**<0.001** ^1^	65 (56, 73)	**<0.001** ^1^
	ALS-specific	74 (71, 77)	82 (81, 84)	**<0.001** ^1^	51 (48, 59)	**<0.001** ^1^
	ALS-nonspecific	26 (25, 27)	30 (30, 31)	**<0.001** ^1^	14 (11, 16)	**<0.001** ^1^
ALS-specific cutoff, n (%)				**0.001** ^2^		**<0.001** ^2^
	Cognitive impairment	17 (24%)	4 (5%)		6 (100%)	
	No cognitive impairment	54 (76%)	81 (95%)		0	
ALS-nonspecific cutoff, n (%)				**<0.001** ^2^		**0.002** ^2^
	Cognitive impairment	22 (31%)	5 (6%)		6 (100%)	
	No cognitive impairment	49 (69%)	80 (94%)		0	
ECAS total cutoff, n (%)				**<0.001** ^2^		**0.002** ^2^
	Cognitive impairment	23 (32%)	4 (5%)		6 (100%)	
	No cognitive impairment	48 (68%)	81 (95%)		0	

Abbreviations: AD, Alzheimer’s disease; ALS, amyotrophic lateral sclerosis; CI, confidence interval; ECAS-N, Edinburgh Cognitive and Behavioural ALS screen-translated Norwegian version; HC, healthy controls

^1^t-test for independent groups

^2^chi-square test for independence.

**Fig 4:**
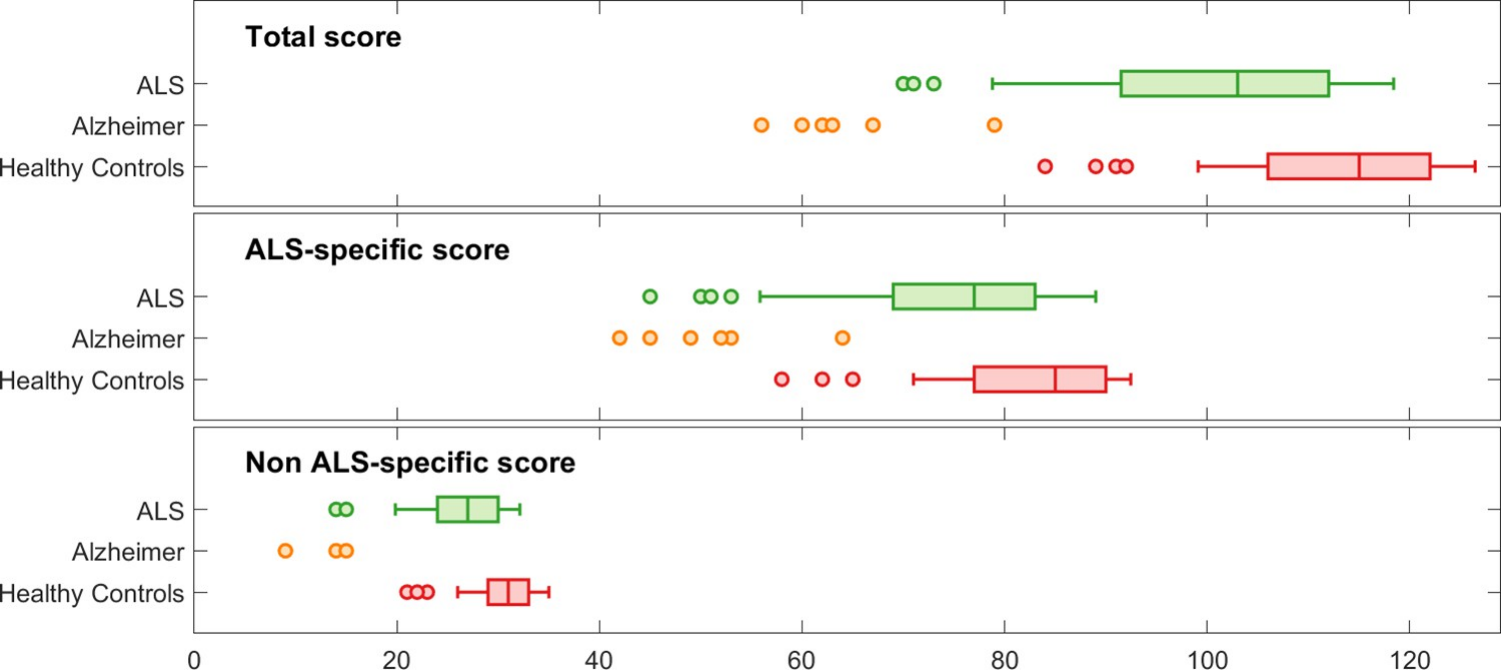
Descriptive visualization of ECAS-N total scores, ALS-specific scores and ALS-nonspecific scores (median, min, max) for subjects with ALS, controls with Alzheimer’s disease and Healthy Controls, testing hypotheses 2–5.

In the testing of H3 and H4, we found that ECAS-N ALS-specific score, ALS-nonspecific score and cutoff scores in subjects with ALS were significantly better than those of controls with AD (Table 4), supporting H3 and rejecting H4. In testing of H5, we found that ECAS-N total scores, ALS-specific score, ALS-nonspecific score and cutoff scores in subjects with ALS were significantly better than those controls with AD (Table 4), supporting H5. The association were however, higher than expected, as we initially expected that the ALS-specific score and ALS-nonspecific score would diverge in different directions. Fig 4 presents a visualization of the results of tests of H2-H5.

## 4. Discussion

Mortality and morbidity related to ALS is becoming more of a public health issue within the Norwegian aging population [50]. Thus, there is a great need to have a valid, trustworthy Norwegian language version of the ECAS (ECAS-N). With this multicentre study, we filled this gap and presented the first results on psychometrics of the ECAS-N. We found that the internal consistency of the ECAS-N differs from that reported for the original ECAS. ECAS-N had excellent inter-rater reliability. The test-retest reliability was acceptable for ECAS-N total score, ALS specific-score and behavioural score, however, Bland Altman plot indicated a potential bias. Evaluation of construct validity suggested that the ECAS-N and MoCA assess similar, but not the same construct, and that the ECAS-N could be used to distinguish people with ALS-specific cognitive impairment from HCs and those with AD. Discussion of several findings from our study will aid clinicians and researchers who want to routinely use the ECAS-N.

Of a total sample of 71 ALS subjects in our study -none of whom had comorbidities of importance related to cognitive function-24% scored below the cutoff on the ALS-specific ECAS-N. According to revised consensus criteria of Strong et al., these subjects are ALS patients with cognitive impairment [9]. This 24% prevalence rate is slightly below the rate of abnormal performance in ALS previously reported when using the original ECAS (29%) and the 30% rate derived from a meta-analysis of studies that used different but commonly used neuropsychological tests for diagnosis [1, 27]. These differences may be explained by individual differences in the studies, or different cutoff values used in different versions of the ECAS. Based on 69 interviews with carers, we found 30% of our sample met the Strong criteria of ALSbi [9]. This is close to the median prevalence (25%) of mild-to-moderate behavioral changes reported in a systematic review [2].

Our finding of apathy as the most frequently reported behavioral change in our sample of ALS patients is in line with what Abrahams et al. reported for the original ECAS [27], while Raaphorst et al. found the most frequently reported behavioral change was perseveration, followed by apathy [2]. Our findings indicated that 3% of the ALS subjects met the criteria of ALS-FTD, a result slightly below the prevalence reported in a systematic review (8%) and that found when using the original ECAS (6%) [2, 27].

### 4.1. Internal consistency

Our analysis of internal consistency of the ECAS-N produced a Cronbach’s alpha of 0.72 for the behavioral scale and 0.65 for the total ECAS scale. These results point to the importance of considering both scores instead of one or the other in isolation. The result for total score is slightly below the common acceptable alpha value (α = 0.70) [51].

Interpretation of Cronbach’s alpha is not straightforward. At worst, our finding of a Cronbach’s α = 0.65 may temper use of the ECAS-N cognitive screen at this time. Alternatively, the screen might be a measure of two different aspects of cognition (ALS-specific and ALS-nonspecific), which would mean that the internal consistency analysis of total score is not relevant [26]. The alpha of the original total ECAS scale was α = 0.75, and it was indicated that the sub-scores of the original ECAS correlate well with the total score [27]. However, information about the scales unidimensionality, a prerequisite for a clear interpretation of the internal consistency statistics [26, 51], was not provided.

We found a higher internal consistency Cronbach’s alpha for the ALS-specific score (α = 0.62) than for ALS-nonspecific score (α = 0.33), meaning that the ALS-specific score dominated the ECAS-N total score. The high levels of content validity previously reported for all items of the ECAS-N [24] support the notion that the ECAS-N validly taps into underlying constructs of cognition and behavior among respondents, allowing its use in clinical and research settings.

### 4.2. Inter-rater reliability

The ECAS-N exhibited excellent inter-rater reliability. This is an important outcome because it allows various testers trained in the use of ECAS to administer the test and produce comparable results. Ideally, we suggest testers obtain certification through the online certification system for ECAS training (https://ecas.psy.ed.ac.uk/training/).

### 4.3. Test-retest reliability

The results of our test-retest analysis of the ECAS-N is relevant only for a test-retest interval of four months, as that is the period we assessed. The observed differences revealed by Bland-Altman plots indicated a bias between the mean differences. However, the trend was not clear, and it is difficult to interpret whether the observed differences in test performance was due to a change in the patients’ illness or problems with the instrument itself. Based on available research, we assumed recall bias would probably not occur in patients with ALS [36, 37]. However, a practice effect has been demonstrated for the original ECAS in healthy control persons at a six-month test-retest interval [36]. This suggests ALS patients in our study who have no cognitive impairment may have improved on the second administration of the ECAS-N due to their experience with the same test version four months earlier. In HCs, using alternate forms of the ECAS seems to mitigate a practice effect in learning test content or adopting test-taking strategies [52]. This means that translation of alternate forms of the ECAS into Norwegian is warranted.

We relied on research suggesting cognitive changes emerge before motor symptoms and remain stable after an initial decline [53]. Our results, however, may support those who claim that cognitive deficits become more prominent over time, especially early in the course of disease [54].

### 4.4. Construct validity

We found a moderate positive (r = 0.53) correlation between the scores on the ECAS-N and the MoCA for persons with ALS. This is not surprising when comparing an ALS-specific test with an ALS-nonspecific test, and this has been observed for other translations of the ECAS [17, 21]. Identifying more subjects classified as impaired by the MoCA than by the ECAS-N may simply be because the MoCA is a more sensitive test for detecting mild cognitive impairment [55].

Further analysis of construct validity in the present study suggested that the ECAS-N may be used to validly distinguish persons with ALS-specific cognitive impairment from those with no cognitive impairment and those who have cognitive impairment due to AD. However, we included a small number of subjects with AD in our analysis, which limits what conclusions can be drawn.

### 4.5. Study strength and limitations

Our study had several strengths. Designing a multicenter study involving most of the large hospitals in Norway increases the probability that our sample was reasonably representative of Norwegian patients with ALS. Another strength is that we used experienced and highly trained testers, many of whom were officially certified for administering the ECAS. Employing the expertise of a biostatistician to guide our selection of appropriate statistical methods to use also was a strength. We have been transparent about our methodological choices, meaning others can easily evaluate the methodological quality of our study.

Readers must be aware that the sample size were not statistically estimated *a-priory*, but relied on a fixed analysis and recommendations in the litterature [39]. Although this is a limitation of the study, a post-hoc power analysis is meaningless. Significant p-values, indicate that we had enough power to detect differences in our hypothesis testing. However, a possible bias might be present related to a limited sample size in some of the other analysis; smaller sample size typically mean low statistical power. The baseline characteristics (Table 2), were however, well balanced between the ALS subjects included in the different analysis. In an attempt to prevent recall bias, we chose a four-month time interval between test and retest. Based on previous research, we assumed that the subjects with ALS were cognitively stable in the interim period [36]; however, we cannot be certain. Information about subjects’ respiratory support, stress level, and lack of sleep might have made it easier to interpret results of the ECAS-N. In the real-world setting, it was not reasonable to try to completely control all subjects and environmental conditions when we tested subjects. However, this factor is important for testers to be cognizant of, as the effect of the environment on ECAS-N results is not fully understood.

## 5. Conclusions

It is critical to rigorously evaluate the psychometric soundness of translated neurobehavioral and cognitive screening tests, since the translation process itself and adjustments for culture differences in the target language may threaten essential features of the original validated instrument. Internal consistency of the ECAS-N differs from that reported for the original ECAS. However, high levels of content validity still give reasons to argue that the two versions’ equivalency is maintained. The ECAS-N seems not to be affected by who administers the test, permitting its valid use by various clinical practitioners. Trustworthiness that the ECAS-N scores are stable between four and eight months is fragile, and results from repeated assessments must be interpreted with care. Further evidence to support the construct validity of the ECAS has been generated. It seems likely that the ECAS-N and MoCA measure similar, but not the same construct. The ECAS-N seems to distinguish people with ALS-specific cognitive impairment from people with no cognitive impairment and those who have cognitive impairment due to AD.

## 6. Considerations for future studies

Further research will contribute to a more robust documentation of the psychometric properties of the ECAS-N. In future studies, the authors should consider conducting *a-priori* power analysis to determine an appropriate sample size. Investigating the test-retest reliability at various intervals will provide more information about the ideal interval for assessing cognitive ability in patients with ALS. Gathering information about subjects’ respiratory support, stress level, and sleep could help in interpreting the results and controlling for potential confounding factors. To draw more reliable conclusions about the ECAS-N’s ability to distinguish between ALS patients and those with Alzheimer’s Disease (AD), the sample size of subjects with AD should be appropriate. To mitigate potential practice effects there is a need to translate alternate forms of the ECAS into Norwegian and assessing their psychometric properties. Within the field of ALS there is also a need to better understand how environmental factors may affect test’s performance, such there is also a need to examine the influence of different environmental factors on the ECAS-N results.

## Supporting information

S1 Fig Bland-Altman results for inter-rater agreement of each domain of the ECAS-N (top row), and Bland-Altman plot for each domain of the ECAS-N scores at test time 1 and time 2 (bottom row). The middle solid line represents the mean difference between ECAS-N scores at time 1 and time 2. The lower and upper dashed lines represents the upper and lower 95% confidence limits, respectively.
(TIF)

S1 Table Internal consistency, inter-rater, and test-retest reliability of the ECAS-N cognitive screen in subjects with ALS. (DOCX)
