# Supplementary figures and images for: Norwegian version of the Edinburgh cognitive and behavioural ALS screen: Construct validity, internal consistency, inter-rater, and test-retest reliability
Source: PLoS One. 2023 May 4;18(5):e0285307. doi: 10.1371/journal.pone.0285307 (PMC10159149; doi:10.1371/journal.pone.0285307)

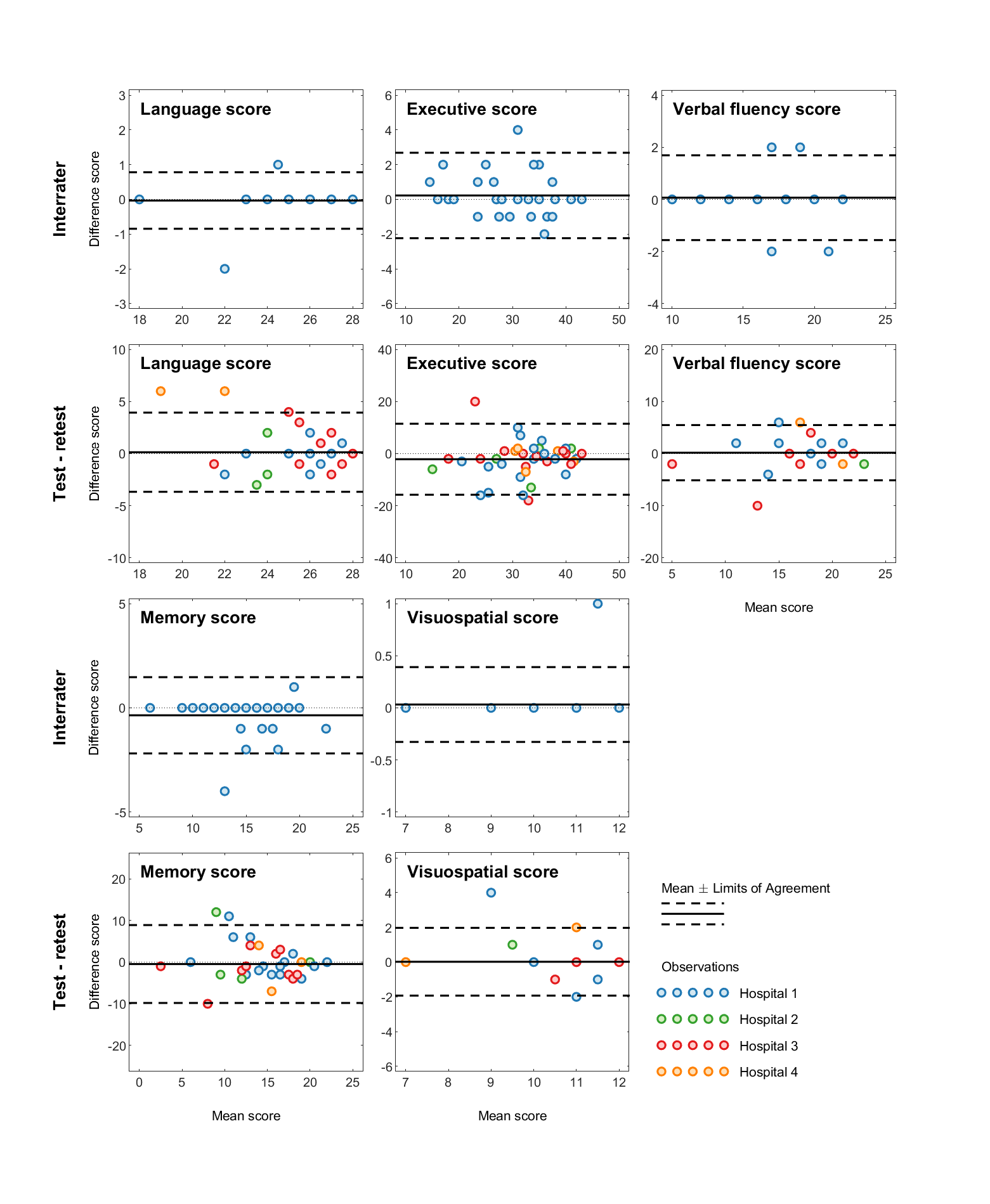

Supplement: S1 Fig — Bland-Altman results for inter-rater agreement of each domain of the ECAS-N (top row), and Bland-Altman plot for each domain of the ECAS-N scores at test time 1 and time 2 (bottom row). The middle solid line represents the mean difference between ECAS-N scores at time 1 and time 2. The lower and upper dashed lines represents the upper and lower 95% confidence limits, respectively. (TIF) [file pone.0285307.s001.tif]
