# Supplementary material for: Norwegian version of the Edinburgh cognitive and behavioural ALS screen: Construct validity, internal consistency, inter-rater, and test-retest reliability
Source: PLoS One. 2023 May 4;18(5):e0285307. doi: 10.1371/journal.pone.0285307 (PMC10159149; doi:10.1371/journal.pone.0285307)
Supplement: S1 Table — (DOCX) [file pone.0285307.s002.docx]

**S1 Table.** Internal consistency, inter-rater, and test-retest reliability of the ECAS-N cognitive screen in subjects with ALS

|  | | | **Internal consistency** | | **Inter-rater reliability** | | | **Test-retest reliability** | | |
| --- | --- | --- | --- | --- | --- | --- | --- | --- | --- | --- |
| ECAS-N (number of items) | | | n | Cronbach’s alpha | n | ICC^1^  Cohen’s kappa | (95% CI) | n | ICC^2^  Cohen’s kappa | (95% CI) |
| Cognitive screen | | |  |  |  |  |  |  |  |  |
|  | Total score (15) | | 71 | 0.65 | 31 | 0.99 | (0.98, 1.00) | 47 | 0.73 | (0.56, 0.84) |
|  | ALS-specific score (9) | | 71 | 0.62 | 31 | 0.99 | (0.98, 1.00) | 47 | 0.72 | (0.54, 0.83) |
|  |  | Language score (3) | 71 | 0.07 | 31 | 0.98 | (0.96, 0.99) | 47 | 0.63 | (0.42, 0.77) |
|  |  | Verbal fluency score (2) | 71 | 0.62 | 31 | 0.97 | (0.93, 0.98) | 47 | 0.72 | (0.54, 0.83) |
|  |  | Executive score (4) | 71 | 0.37 | 31 | 0.99 | (0.98, 0.99) | 47 | 0.57 | (0.34, 0.74) |
|  | ALS-nonspecific score (6) | | 71 | 0.33 | 31 | 0.97 | (0.94, 0.99) | 47 | 0.61 | (0.39, 0.76) |
|  |  | Memory score (3) | 71 | 0.39 | 31 | 0.97 | (0.93, 0.98) | 47 | 0.60 | (0.39, 0.76) |
|  |  | Visuospatial score (3) | 71 | 0.13 | 31 | 0.99 | (0.98, 0.99) | 47 | 0.57 | (0.34, 0.74) |
| Abbreviations: ALS, amyotrophic lateral sclerosis; ECAS-N, Edinburgh Cognitive and Behavioural ALS screen-translated Norwegian version; ICC, Intraclass correlation coefficient; SD, Standard deviation. ^1^Two-way random-effect analysis of variance model with interaction term for the absolute agreement between single raters; ^2^Two-way mixed-effect analysis of variance model with interaction term for the absolute agreement between single measurement. | | | | | | | | | | |
